# Tyrosine Kinase Inhibitors Outperform Immune Checkpoint Inhibitors in Bone-Predominant Metastatic Renal Cell Carcinoma: A Multicenter Real-World Analysis

**DOI:** 10.7150/jca.113258

**Published:** 2025-09-22

**Authors:** Lingbin Meng, Sarah P. Psutka, Jinesh Gheeya, Mingjia Li, Meghana Noonavath, Delaney Orcutt, Evan Gross, Katharine A. Collier, Amir Mortazavi, Edmund Folefac, Paul Monk, Yuanquan Yang

**Affiliations:** 1 Division of Medical Oncology, Department of Internal Medicine, The Ohio State University Comprehensive Cancer Center, Columbus, OH 43210, USA.; 2 Department of Urology, The University of Washington and Fred Hutchinson Cancer Center, Seattle, WA 98195, USA.

**Keywords:** bone-predominant metastatic renal cell carcinoma, tyrosine kinase inhibitors, TKIs, immune checkpoint inhibitors, ICIs, overall survival, real-world multicenter analysis

## Abstract

**Background:** Bone-predominant metastatic renal cell carcinoma (mRCC) presents significant clinical challenges due to its associated morbidities and poor prognosis. Optimal first-line treatment remains unclear, largely because these patients are often excluded from clinical trials due to difficulties in measuring bone lesions. Emerging evidence suggests that bone metastases exhibit high angiogenesis gene signatures, potentially predicting favorable responses to tyrosine kinase inhibitors (TKIs).

**Methods:** We conducted a multicenter retrospective analysis of patients with bone-predominant mRCC treated at The Ohio State University Comprehensive Cancer Center and Fred Hutchinson Cancer Center from January 2008 to June 2021. Bone predominance was defined as having a greater number of osseous metastases compared to extra-osseous sites using computed tomography or bone scans. Patients receiving first-line TKIs or immune checkpoint inhibitors (ICIs) were included; those treated with combination TKI-ICI therapies were excluded due to limited numbers. Demographic, clinical, and treatment data were collected. Progression-free survival (PFS) and overall survival (OS) were analyzed using Kaplan-Meier methods and compared using the log-rank test. Univariate Cox regression analysis was conducted to identify factors associated with OS.

**Results:** A total of 69 patients with bone-predominant mRCC were identified, with 40 receiving TKIs and 29 receiving ICIs as first-line therapy. Baseline characteristics were comparable between groups. The median OS was significantly longer for patients treated with TKIs compared to those receiving ICIs (41.3 months vs. 19.3 months; log-rank *P* = 0.036). A trend toward improved median PFS was observed in the TKI group (7.9 months vs. 4.9 months; *P* = 0.075). Univariate analysis showed that treatment with ICIs was associated with an increased risk of death compared to TKIs (hazard ratio = 1.96; *P* = 0.040). Objective response rates were higher in the TKI group (22.9% vs. 12.0%), although this difference was not statistically significant (*P* = 0.332).

**Conclusions:** In this multicenter real-world analysis, first-line treatment with TKIs was associated with significantly improved OS compared to ICIs in patients with bone-predominant mRCC. These findings suggest that TKI-containing regimens may be the preferred front-line therapy for this patient subgroup. Prospective studies are warranted to validate these results and to optimize treatment strategies for bone-predominant mRCC.

## Introduction

Renal cell carcinoma (RCC) comprises around 90% of kidney malignancies, with metastatic disease affecting approximately 60% of patients - either present at initial diagnosis (30%) or developing during disease progression (30%) 1-3. Among patients with metastatic RCC (mRCC), bone represents one of the most common sites of distant spread, affecting approximately 30-35% of patients 4-6. Bone-predominant mRCC, characterized by a greater burden of osseous than extra-osseous metastases, presents unique clinical challenges 7-9. These patients experience significant morbidity, including pathological fractures, spinal cord compression, and debilitating pain, which substantially impacts their quality of life 10, 11. Moreover, the assessment and measurement of treatment response in bone metastases remains particularly challenging due to their largely non-measurable nature by conventional Response Evaluation Criteria in Solid Tumors (RECIST) 12, 13. This not only complicates clinical decision-making but has historically led to the exclusion of these patients from many pivotal clinical trials, resulting in limited evidence to guide optimal treatment selection 14, 15.

The therapeutic landscape for mRCC has evolved dramatically over the past two decades, transitioning from cytokine-based therapies to more targeted and immunological approaches 16-20. Tyrosine kinase inhibitors (TKIs), which target the vascular endothelial growth factor (VEGF) pathway, emerged as the first major breakthrough, with agents such as sunitinib demonstrating significant improvements in progression-free survival (PFS) (11 months vs. 5 months with interferon-alfa [IFN-α]) as first-line therapy 21. More recently, immune checkpoint inhibitors (ICIs) have revolutionized mRCC treatment, either as monotherapy or in combination with other agents 22-24. The combination of nivolumab plus ipilimumab has shown remarkable efficacy with a median overall survival (OS) of 47 months and a 42% objective response rate (ORR) in intermediate or poor-risk patients 25, 26. These impressive outcomes have led current treatment guidelines to recommend ICI-based combinations as preferred first-line options for patients with intermediate- or poor-risk disease, while TKI monotherapy remains a standard option for favorable-risk patients 27.

Emerging biological insights suggest that bone metastases in RCC exhibit distinct characteristics that may influence treatment outcomes 8, 28. Genomic analyses have revealed that bone metastases typically display elevated angiogenesis gene signatures compared to other metastatic organ systems, with the hypoxia-induced factor (HIF) signaling pathway playing a key role in these processes through upregulation of several growth factors including VEGF, platelet-derived growth factor (PDGF), and transforming growth factor-alpha (TGF-α) 29, 30. Recent molecular analyses have further demonstrated that bone metastases are enriched for polybromo 1 (PBRM1) and tumor protein p53 (TP53) mutations, predominantly express an angiogenic/stromal molecular subtype, and exhibit a distinct microenvironment characterized by increased endothelial cells and monocytic lineages 31. This biological rationale is supported by preliminary clinical observations showing favorable responses to TKIs in patients with bone metastases, as these agents not only target tumor vasculature but may also modulate the bone microenvironment through effects on osteoclasts and osteoblasts 32, 33.

Recent clinical evidence reflects the complexity of optimal treatment selection in this population. A multi-institutional retrospective study demonstrated superior objective response rates with second-line TKIs compared to nivolumab in bone lesions 34, and studies of ICI combinations in mRCC found that patients with a higher burden of bone metastases demonstrated worse disease control rates (DCR) 35 and OS 9. However, challenging these findings, a study of 98 mRCC patients suggested that bone metastases did not significantly impact OS in patients receiving ICI therapy 36, indicating that traditional assumptions about poor prognosis may need to be reevaluated in the immunotherapy era. Despite the improved survival times with modern therapies making optimal treatment selection increasingly crucial, robust real-world evidence directly comparing the efficacy of first-line TKIs versus newer immunotherapy approaches in bone-predominant disease remains limited.

To address this critical knowledge gap, we conducted a multicenter retrospective analysis comparing the effectiveness of first-line TKIs versus ICIs in patients with bone-predominant mRCC. By leveraging real-world data from two major academic cancer centers spanning over a decade, our study aimed to provide clinically relevant evidence to guide treatment selection for this challenging patient population.

## Patients and Methods

### Study population and treatment

**Figure 1:**
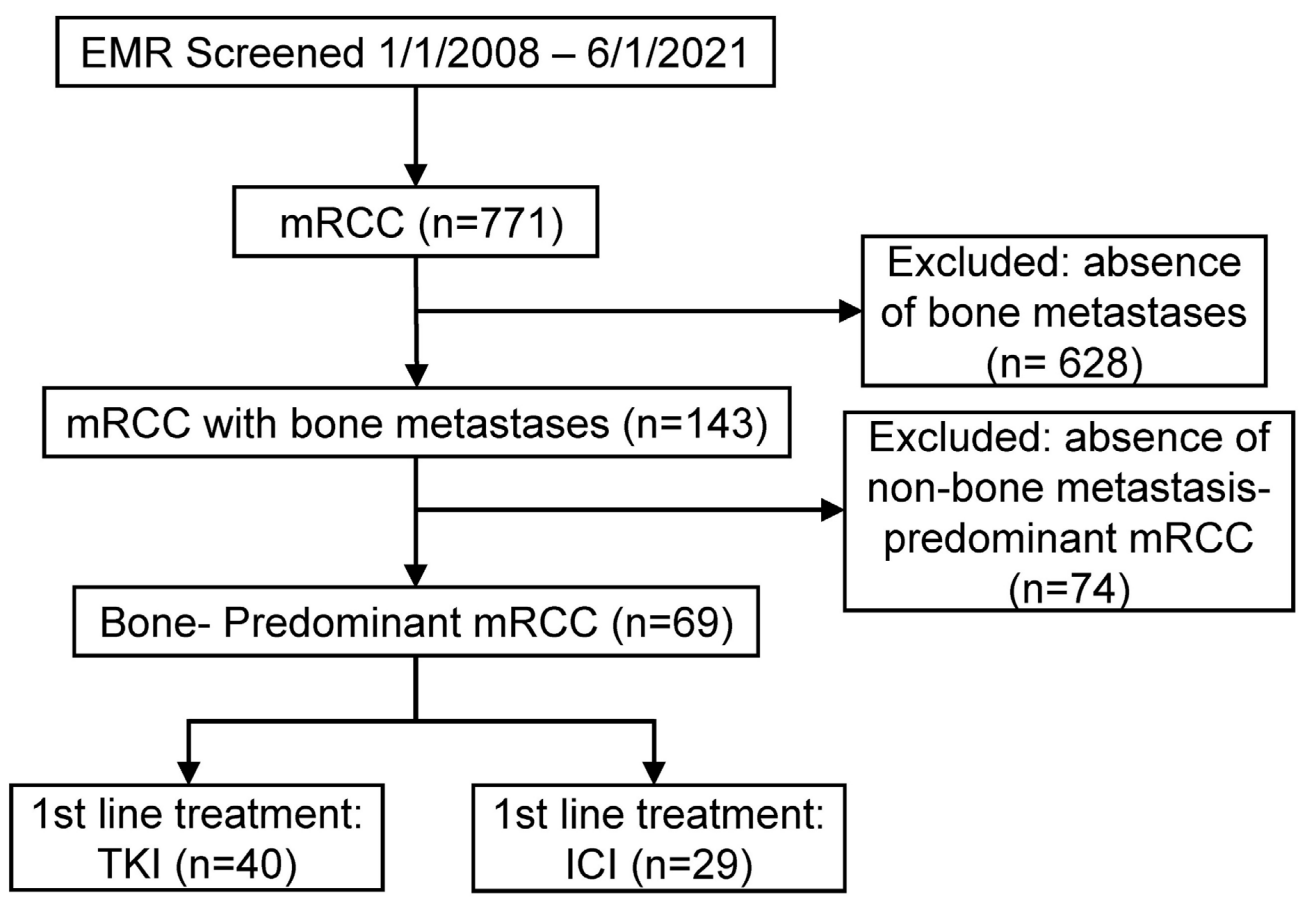
CONSORT diagram for patient selection in the retrospective cohort analysis. Abbreviations: EMR - electronic medical records; mRCC - metastatic renal cell carcinoma; TKI - tyrosine kinase inhibitor; ICI - immune checkpoint inhibitor.

We conducted a retrospective analysis of patients diagnosed with mRCC at The Ohio State University Comprehensive Cancer Center and the University of Washington Fred Hutchinson Cancer Center between January 2008 and June 2021. As shown in **Figure 1**, a total of 771 patients with mRCC were initially screened. Among these, 143 patients had bone metastases, and 69 patients were identified as having bone-predominant disease, defined as a greater number of osseous metastases compared to extra-osseous metastases. These 69 patients were included in the study. Patients were stratified into two groups based on their first-line systemic therapy: those who received TKIs and those who received ICIs. The TKI group comprised 40 patients, while the ICI group included 29 patients. Patients who received combination therapies of TKIs and ICIs were excluded due to limited numbers. First-line TKIs administered included sunitinib, pazopanib, cabozantinib, sorafenib, axitinib, and tivozanib. In the ICI group, patients received either single-agent ICIs (nivolumab or pembrolizumab) or combination ICI therapy with nivolumab plus ipilimumab (**Supplemental Table 1**). This study was approved by the Institutional Review Boards of both participating institutions. Due to the retrospective nature of the study, the requirement for informed consent was waived.

### Study design and assessments

Baseline demographic and clinical data were collected from electronic medical records, including age, gender, histology, Eastern Cooperative Oncology Group (ECOG) performance status, International Metastatic RCC Database Consortium (IMDC) risk category, prior nephrectomy status, and the number of metastatic organ systems. Treatment details, subsequent therapies, and responses were also recorded. Treatment responses were evaluated using RECIST version 1.1. ORR was defined as the proportion of patients achieving a complete response (CR) or partial response (PR). DCR included patients who achieved CR, PR, or stable disease (SD). PFS was defined as the time from the initiation of first-line therapy to documented disease progression or death from any cause. OS was defined as the time from the initiation of first-line therapy to death from any cause.

### Statistical analysis

Statistical analyses were performed using SPSS Biostatistics 27. Baseline characteristics were compared between the TKI and ICI groups using Fisher's exact test for categorical variables and the Mann-Whitney U test for continuous variables. Survival analyses for PFS and OS were conducted using the Kaplan-Meier method and compared with the log-rank test. A univariate Cox proportional hazards model was employed to assess the association of clinical variables with OS. Variables included in the model were treatment type (ICI vs. TKI), age, gender, histology (clear cell vs. non-clear cell), ECOG performance status, IMDC risk category, prior nephrectomy, and the number of metastatic organ systems. Response rates were compared using Fisher's exact test. All tests were two-sided, and a *P*-value of less than 0.05 was considered statistically significant.

## Results

### Demographics and clinical characteristics of bone-predominant mRCC patients

A total of 69 patients with bone-predominant mRCC were included in the analysis, with 40 patients receiving TKIs and 29 patients receiving ICIs as first-line therapy. The baseline demographic and clinical characteristics of these patients are summarized in **Table 1**. The median age was similar between the TKI and ICI groups (64.5 vs. 64 years, respectively; *P* = 0.422). The majority of patients were male (71.0%) and had clear cell histology (78.3%), with no significant differences between the treatment groups for gender (*P* = 0.592) or histology (*P* = 0.690). Most patients had an ECOG performance status of 0 or 1 (66.7%) and were classified as intermediate risk according to the IMDC criteria (58.0%), with comparable distributions across both groups. Notably, a higher proportion of patients in the TKI group had undergone prior nephrectomy compared to the ICI group (67.5% vs. 44.8%), approaching statistical significance (*P* = 0.0842). The number of metastatic organ systems was similar between groups (*P* = 0.429), with approximately 71.0% of patients having only one metastatic site (bone). Patients in the TKI group received an average of 2.36 subsequent lines of therapy compared to 1.38 in the ICI group (**Supplemental Table 2**). The median follow-up period was 20.6 months (IQR: 14.4-41.6 months) for the entire cohort, with 25.3 months (IQR: 16.5-67.2 months) for the TKI group and 17.3 months (IQR: 13.5-23.5 months) for the ICI group.

Overall, there were no statistically significant differences in baseline characteristics between the TKI and ICI groups, suggesting that the cohorts were comparable for outcome analysis purposes.

### Improved OS and trend toward better PFS in patients treated with TKIs

**Figure 2:**
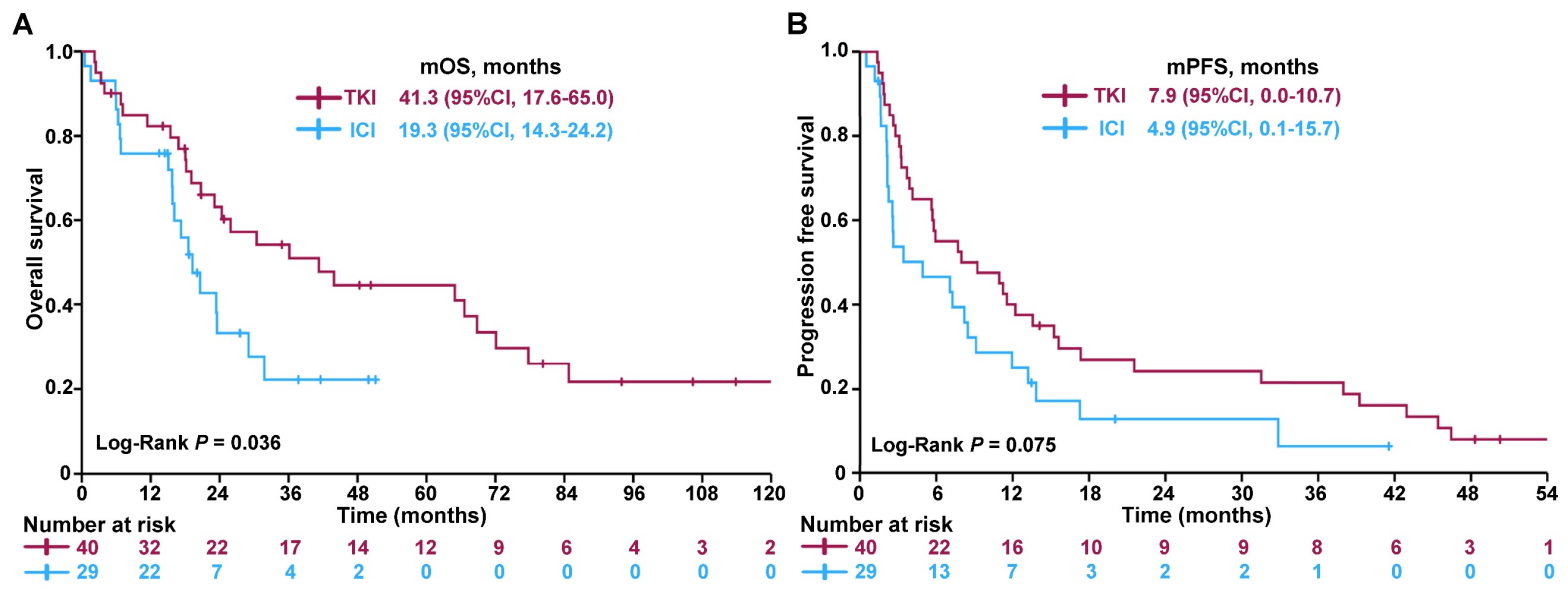
Kaplan-Meier survival curves comparing (A) OS and (B) PFS between patients treated with TKIs and ICIs. Abbreviations: OS - overall survival; PFS - progression-free survival; TKI - tyrosine kinase inhibitor; ICI - immune checkpoint inhibitor; CI - confidence interval.

Patients with bone-predominant mRCC who received TKIs demonstrated significantly improved OS compared to those treated with ICIs. The median OS was 41.3 months for the TKI group versus 19.3 months for the ICI group (**Figure 2A**), with the difference reaching statistical significance (log-rank test, *P* = 0.036). In terms of PFS, there was a trend favoring the TKI group. The median PFS was 7.9 months for patients treated with TKIs compared to 4.9 months for those receiving ICIs (**Figure 2B**). Although this difference did not reach statistical significance (log-rank test, *P* = 0.075), it suggests a potential benefit of TKIs in delaying disease progression.

To identify factors associated with OS, a univariate Cox regression analysis was performed (**Table 2**). Treatment with ICIs (versus TKIs) was associated with a nearly two-fold increased risk of death (hazard ratio [HR] = 1.96; 95% confidence interval [CI]: 1.03-3.71; *P* = 0.040). Additionally, non-clear cell histology was significantly associated with worse OS (HR = 0.30; 95% CI: 0.13-0.74; *P* = 0.009), indicating that patients with clear cell carcinoma had better survival outcomes. Other variables, such as age, gender, ECOG performance status, IMDC risk category, prior nephrectomy, and the number of metastatic organ systems, were not significantly associated with OS in this analysis.

These findings suggest that first-line treatment with TKIs may confer a survival advantage over ICIs in patients with bone-predominant mRCC and highlight the importance of histological subtype in predicting patient outcomes.

### Trend toward higher ORR and DCR with first-line TKIs

In patients with bone-predominant mRCC, first-line treatment with TKIs demonstrated a trend toward higher ORR and DCR compared to ICIs, although these differences were not statistically significant. Among the evaluable patients (*n*=60), none in either treatment group achieved CR. PR was observed in 22.9% of patients treated with TKIs (8/35) compared to 12.0% in the ICI group (3/25). SD was achieved by 37.1% of TKI-treated patients and 40.0% of ICI-treated patients. Progressive disease (PD) occurred in 40.0% of patients receiving TKIs and 48.0% of those receiving ICIs.

**Figure 3:**
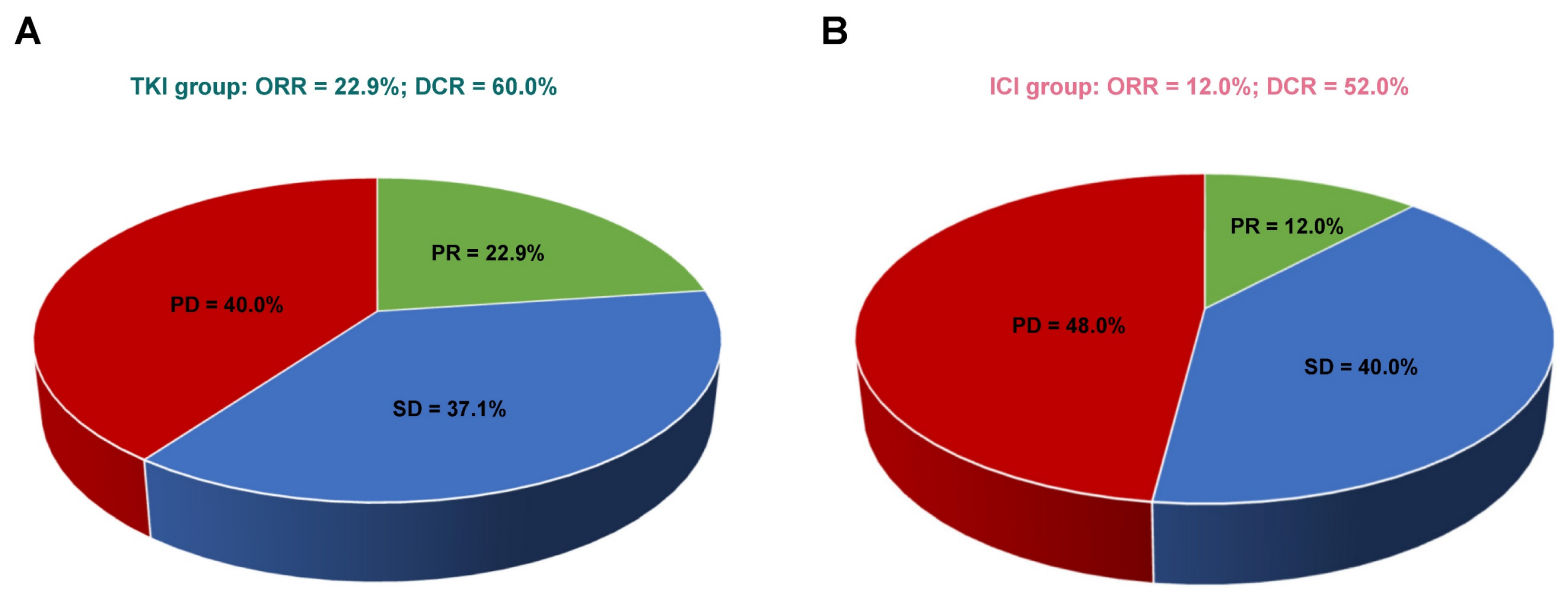
Comparison of response rates in patients receiving TKIs versus ICIs. (A) Response rates in the TKI group. (B) Response rates in the ICI group. Abbreviations: CR - complete response; PR - partial response; SD - stable disease; PD - progressive disease; ORR - objective response rate; DCR - disease control rate.

The ORR was higher in the TKI group (22.9%) compared to the ICI group (12.0%), but this difference did not reach statistical significance (*P* = 0.332). The DCR was also higher in the TKI group (60.0%) versus the ICI group (52.0%), with no statistically significant difference (*P* = 0.603). **Figure 3** illustrates the comparison of response rates between the two treatment groups. While the data suggest a potential advantage of TKIs in achieving tumor response and disease control, the lack of statistical significance indicates that larger studies are needed to confirm these trends.

## Discussion

Bone-predominant mRCC poses significant treatment challenges, and the optimal first-line therapy for this subgroup has not been well established 7-9, 28. While ICIs have shown promising results in mRCC overall 22-24, their effectiveness in bone-predominant disease remains uncertain due to limited evidence 9, 35, 36. In this multicenter retrospective study, we evaluated the efficacy of first-line TKIs compared to ICIs in patients with bone-predominant mRCC. Our findings revealed a significant improvement in OS with TKI therapy, showing a median OS of 41.3 months versus 19.3 months with ICIs (*P* = 0.036). Additionally, there were trends toward enhanced PFS, ORR, and DCR in the TKI group, although these differences did not reach statistical significance. Univariate analysis confirmed the survival advantage of TKI treatment (HR 1.96 for ICI vs. TKI, *P* = 0.040) and identified clear cell histology as a significant prognostic factor (HR 0.30, *P* = 0.009). These results offer valuable insights into the potential benefits of TKI therapy over ICIs for managing bone-predominant mRCC.

The superior OS observed with TKIs in bone-predominant mRCC may be attributed to the unique biological characteristics of bone metastases. Bone lesions in RCC are known to exhibit high angiogenesis gene signatures, including upregulation of VEGF, PDGF, and TGF-α 29, 30. This angiogenic phenotype is further supported by the recent molecular analysis by Gulati et al. 31, which demonstrated that bone metastases have distinct genomic and transcriptomic features, with significant enrichment of PBRM1 mutations (59.6%) and TP53 mutations (22.9%). Notably, bone metastases showed higher expression of PDL1 and PDL2 compared to primary kidney tumors and were predominantly characterized by the angiogenic/stromal molecular subtype. The study also revealed that bone metastases had a greater abundance of endothelial cells and cells of monocytic lineage in their microenvironment, along with increased fibroblast populations. These molecular features, particularly the angiogenic/stromal signature, may explain the enhanced efficacy of TKIs in bone metastases. Moreover, TKIs may exert additional effects within the bone microenvironment. They can modulate bone remodeling by inhibiting osteoclast-mediated bone resorption and affecting osteoblast function 32, 33. The study's finding of increased expression of extracellular matrix reorganization genes (including ASPN, DCN, COL6A3, and COL11A1) in bone metastases suggests active remodeling of the bone microenvironment, which may be particularly susceptible to TKI intervention 31, 37. The potential synergy between TKIs and bone-targeted therapies, such as bisphosphonates or denosumab, could further enhance therapeutic efficacy, although this warrants further investigation.

The relatively lower efficacy of ICIs compared to TKIs in patients with bone-predominant mRCC observed in our study may be attributed to the immunologically “cold” nature of bone metastases 38, 39. The bone marrow microenvironment is enriched with immunosuppressive cells, such as regulatory T cells (Tregs) and myeloid-derived suppressor cells (MDSCs), which inhibit effective antitumor immune responses 40-42. Additionally, limited infiltration of cytotoxic T lymphocytes (CTLs) into bone lesions may further reduce the effectiveness of ICIs, which depend on reinvigorating the host immune system to combat tumor cells 43-45. This biological rationale aligns with clinical observations from prior studies. Clinical research examining ICI combination therapies in mRCC has demonstrated an inverse relationship between bone metastatic burden and treatment outcomes, with higher bone involvement correlating with reduced DCR 35 and OS 9. However, a multicenter study by Gambale et al., which analyzed 98 mRCC patients treated with ICIs, reported no statistically significant difference in OS between patients with and without bone metastases (*P* = 0.254) 36. This discrepancy likely stems from differences in study populations; the Gambale et al. cohort included patients with mixed metastatic patterns and lower bone-specific burden, while our study focused on bone-predominant disease. Furthermore, the Gambale study suggested that factors such as neutrophil-to-lymphocyte ratio (NLR) and basal calcemia levels significantly influenced OS in patients with bone metastases, underscoring the complex interplay between systemic inflammatory markers and treatment outcomes 36.

Our findings carry important clinical implications for managing bone-predominant mRCC. The significant OS benefit observed with TKIs suggests that TKI-containing front-line therapy may be the preferred treatment option for this patient subgroup. This is particularly relevant given that current guidelines often recommend ICI-based combinations as first-line therapy for intermediate- and poor-risk patients 27. Notably, potential confounding factors such as prior nephrectomy and histology do not appear to explain the OS advantage observed with TKIs. While a higher proportion of patients in the TKI group underwent nephrectomy (67.5% vs. 44.8%), univariate analysis did not support nephrectomy as a significant factor for improved OS (*P* = 0.288). Nonetheless, we recognize that cytoreductive surgery may offer survival benefits in well-selected patients and could be subject to selection bias (i.e., patients with better performance status and fewer metastatic sites may be more likely to undergo nephrectomy). Future prospective studies are needed to clarify whether nephrectomy confers additional benefit in this subgroup. Similarly, although clear cell histology was associated with better survival outcomes (HR = 0.30; *P* = 0.009), the proportion of clear cell carcinoma was slightly lower in the TKI group (77.5% vs. 79.3%). A brief descriptive review of our data indicated that clear cell tumors generally had longer OS in both arms, but definitive subgroup analyses were limited by the modest sample sizes. These findings suggest that the observed OS benefit is likely attributable to the initial choice of therapy rather than these variables. Additionally, patients in the TKI group received more subsequent lines of therapy on average compared to the ICI group (2.36 vs. 1.38), as shown in Supplemental Table 2, which might have contributed to the improved OS. However, the univariate analysis did not show that prior nephrectomy or the number of metastatic organ systems significantly affected survival, reinforcing that the initial choice of therapy plays a crucial role in patient outcomes.

Our study has several strengths and limitations that warrant discussion. The key strengths include its multicenter design and use of real-world data from two major academic centers, enhancing the generalizability of our findings. By focusing on the specific population of bone-predominant mRCC—a group often excluded from clinical trials—we address a critical gap in the literature. The long follow-up period allows for a comprehensive assessment of survival outcomes. However, important limitations should be considered when interpreting our results. The retrospective nature introduces potential selection biases and limits the ability to establish causality. The modest sample size (*n* = 69) may reduce the statistical power to detect significant differences in PFS and response rates and precludes robust multivariate analyses due to the risk of overfitting. Furthermore, the evolving treatment landscape during the study period may also affect the applicability of our findings. Specifically, we excluded patients who received TKI-ICI combination therapies (due to limited numbers), which is a significant limitation in light of modern practice, where regimens such as axitinib plus pembrolizumab have become first-line standards. As a result, our findings primarily inform the comparison between TKI monotherapy and ICI monotherapy, and it is unclear how they might translate to combination approaches. Prospective studies evaluating TKI-ICI combinations in bone-predominant disease will be essential to determine whether our results extend to current therapeutic regimens. It is also noteworthy that the TKI group had a longer median follow-up (25.3 vs. 17.3 months), which may partially account for the observed OS advantage. Further follow-up of the ICI cohort is necessary to determine whether this survival gap persists over time. Finally, we lacked molecular or advanced imaging data to confirm the angiogenesis signatures or immune profiles of bone metastases, which could have provided mechanistic insights into our clinical observations.

In conclusion, our study suggests that first-line treatment with TKIs offers a significant survival advantage over ICIs in patients with bone-predominant mRCC. These findings have important implications for treatment selection and highlight the need for personalized approaches based on metastatic patterns and tumor biology. Until prospective data are available, clinicians should consider TKI-containing regimens as the preferred front-line option for this patient population. Further research is essential to validate these results and to optimize therapeutic strategies for bone-predominant mRCC.

## Supplementary Material

Supplementary tables.

## Figures and Tables

**Table 1:** Demographics and clinical characteristics of patients with bone-predominant mRCC stratified by treatment groups.

	Pts treated with TKI (n=40)	Pts treated with ICI (n=29)	*P* value	All patients (n=69)
Age (Median, IQR), years	64.5 (58.5-73.25)	64 (54-72)	0.422	64 (55-73)
Gender, n (%)			0.592	
Male	27 (67.5)	22 (75.9)		49 (71.0)
Female	13 (32.5)	7 (24.1)		20 (29.0)
Histology, n (%)			0.690	
Clear cell	31 (77.5)	23 (79.3)		54 (78.3)
Non clear cell	5 (12.5)	2 (6.9)		7 (10.1)
Unknown	4 (10.0)	4 (13.8)		8 (11.6)
ECOG, n (%)			0.770	
0/1	27 (67.5)	19 (65.5)		46 (66.7)
2/3	8 (20.0)	7 (24.1)		15 (21.7)
Unknown	5 (12.5)	3 (10.4)		8 (11.6)
IMDC, n (%)			0.816	
Favorable	2 (5.0)	1 (3.5)		3 (4.3)
Intermediate	24 (60.0)	16 (55.2)		40 (58.0)
Poor	9 (22.5)	9 (31.0)		18 (26.1)
Unknown	5 (12.5)	3 (10.3)		8 (11.6)
Nephrectomy, n (%)			0.0842	
Yes	27 (67.5)	13 (44.8)		40 (58.0)
No	13 (32.5)	16 (55.2)		29 (42.0)
# of metastatic organ systems, n (%)			0.429	
1	30 (75.0)	19 (65.5)		49 (71.0)
2/3	10 (25.0)	10 (34.5)		20 (29.0)

**Abbreviations:** mRCC - metastatic renal cell carcinoma; TKI - tyrosine kinase inhibitor; ICI - immune checkpoint inhibitor; IQR - interquartile range

**Table 2:** Univariate Cox regression analyses of clinical variables for predicting overall survival.

Variable	HR (95%CI)		*P* value	
Treatment: ICI vs TKI	1.96 (1.03 - 3.71)		0.040	
Age	1.00 (0.98 - 1.02)		0.670	
Gender: male vs female	0.68 (0.37 - 1.26)		0.225	
Clear cell: yes vs no	0.30 (0.13 - 0.74)		0.009	
ECOG: 2/3 vs 0/1	1.44 (0.68 - 3.08)		0.345	
IMDC: poor vs favorable/intermediate	3.20 (0.76 - 13.43)		0.112	
Nephrectomy: yes vs no	0.72 (0.39 - 1.32)		0.288	
# of metastatic organ systems: 2/3 vs 1		1.46 (0.78 - 2.72)		0.233
				

**Abbreviations:** HR - hazard ratio; CI - confidence interval; ICI - immune checkpoint inhibitor; TKI - tyrosine kinase inhibitor; ECOG - Eastern Cooperative Oncology Group; IMDC - International Metastatic RCC Database Consortium

## References

[1] Hsieh JJ, Purdue MP, Signoretti S, Swanton C, Albiges L, Schmidinger M (2017). Renal cell carcinoma. Nature reviews Disease primers.

[2] Bahadoram S, Davoodi M, Hassanzadeh S, Bahadoram M, Barahman M, Mafakher L (2022). Renal cell carcinoma: an overview of the epidemiology, diagnosis, and treatment. G Ital Nefrol.

[3] Nezami BG, MacLennan GT (2024). Clear cell renal cell carcinoma: a comprehensive review of its histopathology, genetics, and differential diagnosis. International Journal of Surgical Pathology.

[4] Zhang J, Cai D, Hong S (2023). Prevalence and prognosis of bone metastases in common solid cancers at initial diagnosis: a population-based study. BMJ open.

[5] Chen S-C, Kuo P-L (2016). Bone metastasis from renal cell carcinoma. International journal of molecular sciences.

[6] Shi D, Zhang R (2023). Long bone metastases of renal cell carcinoma imaging features: case report and literature review. Oncologie.

[7] Gheeya JS, Li M, Xu M, Zimmerman DE, Collier KA, Wang P (2023). Clinical outcomes of patients with bone-predominant metastatic renal cell carcinoma. Journal of Clinical Oncology.

[8] Mansinho A, Nejo P, Leitão T, Casimiro S, Costa L (2021). Management of bone metastases in renal cell carcinoma: bone-targeted treatments, systemic therapies, and radiotherapy. Journal of Cancer Metastasis and Treatment.

[9] Rizzo M, Pezzicoli G, Tibollo V, Premoli A, Quaglini S (2024). Clinical outcome predictors for metastatic renal cell carcinoma: A retrospective multicenter real-life case series. BMC cancer.

[10] Coleman RE (2006). Clinical features of metastatic bone disease and risk of skeletal morbidity. Clinical cancer research.

[11] Zekri J, Ahmed N, Coleman R, Hancock B (2001). The skeletal metastatic complications of renal cell carcinoma. International journal of oncology.

[12] Ko C-C, Yeh L-R, Kuo Y-T, Chen J-H (2021). Imaging biomarkers for evaluating tumor response: RECIST and beyond. Biomarker research.

[13] Hamaoka T, Costelloe C, Madewell J, Liu P, Berry D, Islam R (2010). Tumour response interpretation with new tumour response criteria vs the World Health Organisation criteria in patients with bone-only metastatic breast cancer. British journal of cancer.

[14] Grosinger AJ, Alcorn SR (2024). An Update on the Management of Bone Metastases. Current oncology reports.

[15] Pielkenrood BJ, van der Velden JM, van der Linden YM, Bartels MM, Kasperts N, Verhoeff JJ (2021). Pain response after stereotactic body radiation therapy versus conventional radiation therapy in patients with bone metastases—a phase 2 randomized controlled trial within a prospective cohort. International Journal of Radiation Oncology* Biology* Physics.

[16] Motzer RJ, Hutson TE, Tomczak P, Michaelson MD, Bukowski RM, Rixe O (2007). Sunitinib versus interferon alfa in metastatic renal-cell carcinoma. New England Journal of Medicine.

[17] Gulati S, Labaki C, Karachaliou GS, Choueiri TK, Zhang T (2022). First-line treatments for metastatic clear cell renal cell carcinoma: an ever-enlarging landscape. The oncologist.

[18] Albiges L, Tannir NM, Burotto M, McDermott D, Plimack ER, Barthélémy P (2020). Nivolumab plus ipilimumab versus sunitinib for first-line treatment of advanced renal cell carcinoma: extended 4-year follow-up of the phase III CheckMate 214 trial. ESMO open.

[19] Shah NJ, Bhattacharya R, Ning N, Shinde R, Schmier J, Tan M (2024). Evolving metastatic renal cell carcinoma (mRCC) treatment landscape in the post vascular endothelial growth factor (VEGF) and immune checkpoint inhibitor (IO) setting. Journal of Clinical Oncology.

[20] Angulo JC, Shapiro O (2019). The changing therapeutic landscape of metastatic renal cancer. Cancers.

[21] Motzer RJ, Hutson TE, Tomczak P, Michaelson MD, Bukowski RM, Oudard S (2009). Overall survival and updated results for sunitinib compared with interferon alfa in patients with metastatic renal cell carcinoma. Journal of clinical oncology.

[22] Barata P, Hatton W, Desai A, Koshkin V, Jaeger E, Manogue C (2020). Outcomes with first-line PD-1/PD-L1 inhibitor monotherapy for metastatic renal cell carcinoma (mRCC): a multi-institutional cohort. Frontiers in oncology.

[23] Stühler V, Rausch S, Maas JM, Stenzl A, Bedke J (2021). Combination of immune checkpoint inhibitors and tyrosine kinase inhibitors for the treatment of renal cell carcinoma. Expert Opinion on Biological Therapy.

[24] Lavacchi D, Pellegrini E, Palmieri VE, Doni L, Mela MM, Di Maida F (2020). Immune checkpoint inhibitors in the treatment of renal cancer: current state and future perspective. International journal of molecular sciences.

[25] Tannir N, Albigès L, McDermott D, Burotto M, Choueiri T, Hammers H (2024). Nivolumab plus ipilimumab versus sunitinib for first-line treatment of advanced renal cell carcinoma: extended 8-year follow-up results of efficacy and safety from the phase III CheckMate 214 trial. Annals of Oncology.

[26] Motzer RJ, Tannir NM, McDermott DF, Arén Frontera O, Melichar B, Choueiri TK (2018). Nivolumab plus ipilimumab versus sunitinib in advanced renal-cell carcinoma. New England Journal of Medicine.

[27] Powles T, Albiges L, Bex A, Comperat E, Grünwald V, Kanesvaran R (2024). Renal cell carcinoma: ESMO Clinical Practice Guideline for diagnosis, treatment and follow-up. Annals of Oncology.

[28] Yuasa T, Urakami S, Yamamoto S, Yonese J, Saito K, Takahashi S (2011). Treatment outcome and prognostic factors in renal cell cancer patients with bone metastasis. Clinical & experimental metastasis.

[29] Jones J, Otu H, Spentzos D, Kolia S, Inan M, Beecken WD (2005). Gene signatures of progression and metastasis in renal cell cancer. Clinical cancer research.

[30] Dunn LK, Mohammad KS, Fournier PG, McKenna CR, Davis HW, Niewolna M (2009). Hypoxia and TGF-β drive breast cancer bone metastases through parallel signaling pathways in tumor cells and the bone microenvironment. PloS one.

[31] Gulati S, Barata PC, Elliott A, Bilen MA, Burgess EF, Choueiri TK (2024). Molecular analysis of primary and metastatic sites in patients with renal cell carcinoma. The Journal of Clinical Investigation.

[32] Vandyke K, Fitter S, Dewar AL, Hughes TP, Zannettino AC (2010). Dysregulation of bone remodeling by imatinib mesylate. Blood, The Journal of the American Society of Hematology.

[33] Huang L, Jiang S, Shi Y (2020). Tyrosine kinase inhibitors for solid tumors in the past 20 years (2001-2020). Journal of hematology & oncology.

[34] Yamamichi G, Kato T, Yoshimura A, Tani M, Horibe Y, Liu Y (2025). Efficacy of Second-line Nivolumab Versus Tyrosine Kinase Inhibitors for Renal Cell Carcinoma With Bone Metastases. Anticancer Research.

[35] Moinard-Butot F, Nannini S, Pautas M, Cazzato RL, Virbel G, Martin S (2024). Evaluation of bone response in metastatic renal cell carcinoma treated in first-line with immunotherapy-based combinations. IKCS-EU 2024.

[36] Gambale E, Palmieri VE, Rossi V, Francini E, Bonato A, Salfi A (2023). Bone metastases in renal cell carcinoma: Impact of immunotherapy on survival. Cancer Diagnosis & Prognosis.

[37] Yuan Z, Li Y, Zhang S, Wang X, Dou H, Yu X (2023). Extracellular matrix remodeling in tumor progression and immune escape: from mechanisms to treatments. Molecular cancer.

[38] Chen S, Lei J, Mou H, Zhang W, Jin L, Lu S (2024). Multiple influence of immune cells in the bone metastatic cancer microenvironment on tumors. Frontiers in Immunology.

[39] Liu C, Wang M, Xu C, Li B, Chen J, Chen J (2021). Immune checkpoint inhibitor therapy for bone metastases: specific microenvironment and current situation. Journal of Immunology Research.

[40] Haist M, Stege H, Grabbe S, Bros M (2021). The functional crosstalk between myeloid-derived suppressor cells and regulatory T cells within the immunosuppressive tumor microenvironment. Cancers.

[41] Zhao E, Wang L, Dai J, Kryczek I, Wei S, Vatan L (2012). Regulatory T cells in the bone marrow microenvironment in patients with prostate cancer. Oncoimmunology.

[42] Barcellos-de-Souza P, Gori V, Bambi F, Chiarugi P (2013). Tumor microenvironment: bone marrow-mesenchymal stem cells as key players. Biochimica et Biophysica Acta (BBA)-Reviews on Cancer.

[43] Yang W, Liu S, Mao M, Gong Y, Li X, Lei T (2024). T-cell infiltration and its regulatory mechanisms in cancers: insights at single-cell resolution. Journal of Experimental & Clinical Cancer Research.

[44] Asano Y, Yamamoto N, Demura S, Hayashi K, Takeuchi A, Kato S (2022). The therapeutic effect and clinical outcome of immune checkpoint inhibitors on bone metastasis in advanced non-Small-Cell lung cancer. Frontiers in oncology.

[45] Tsukamoto S, Mavrogenis AF, Masunaga T, Aiba H, Aso A, Honoki K (2024). Response rate specific to bone metastasis of various cancers for immune checkpoint inhibitors: a systematic review. European Journal of Orthopaedic Surgery & Traumatology.

