# Supplementary material for: Tyrosine Kinase Inhibitors Outperform Immune Checkpoint Inhibitors in Bone-Predominant Metastatic Renal Cell Carcinoma: A Multicenter Real-World Analysis
Source: J Cancer. 2025 Sep 22;16(14):4047–54. doi: 10.7150/jca.113258 (PMC12595247; doi:10.7150/jca.113258)
Supplement: Supplementary file 1 — Supplementary tables. [file jcav16p4047s1.pdf]

Supplemental table 1. First-line therapies received by patients with bone-predominant mRCC.

	TKI or ICI	N (%)
**TKI**	Sunitinib	19 (47.5)
	Pazopanib	11 (27.5)
	Tivozanib	1 (2.5)
	Sorafenib	2 (5.0)
	Axitinib	1 (2.5)
	Cabozantinib	6 (15.0)
**ICI**	Nivolumab	8 (27.6)
	Pembrolizumab	1 (3.4)
	Nivolumab + Ipilimumab	20 (69.0)

Supplemental table 2. Subsequent therapies received by patients with bone-predominant mRCC.

Subsequent Treatments	TKI	ICI
**Avg. # of subsequent lines**	2.36	1.38
TKI	21	14
Anti-PD1/L1	22	3
Anti-PD1/L1 + TKI	6	5
Anti-PD1 + CTLA4	9	0
Anti-PD1 + CTLA4 + TKI	2	0
mTOR inhibitor	5	1
mTOR inhibitor + TKI	6	6
Other	2	2
